# EPSTI1 as an immune biomarker predicts the prognosis of patients with stage III colon cancer

**DOI:** 10.3389/fimmu.2022.987394

**Published:** 2022-10-18

**Authors:** Xitao Wang, Wei Cheng, Xingzhi Zeng, Xiaolin Dou, Zhongyi Zhou, Qian Pei

**Affiliations:** ^1^ Department of Hepatobiliary Surgery, Hunan Provincial People’s Hospital, The First Affiliated Hospital of Hunan Normal University, Changsha, China; ^2^ Key Laboratory of Molecular Radiation Oncology Hunan Province, Changsha, China; ^3^ National Clinical Research Center for Geriatric Disorders, Xiangya Hospital, Central South University, Changsha, China; ^4^ Department of General Surgery, Xiangya Hospital, Central South University, Changsha, China; ^5^ Department of General Surgery, The First Affiliated Hospital of Shaoyang University, Shaoyang, China

**Keywords:** colon cancer, immune infiltration, single-cell sequencing, EPSTI1, prognostic marker

## Abstract

**Objective:**

The poor prognosis and heterogeneity of stage III colon cancer (CC) suggest the need for more prognostic biomarkers. The tumor microenvironment (TME) plays a crucial role in tumor progression. We aimed to explore novel immune infiltration-associated molecules that serve as potential prognostic and therapeutic targets.

**Methods:**

TME immune scores were calculated using “TMEscore” algorithm. Differentially expressed genes between the high and low TME immune score groups were identified and further investigated through a protein-protein interaction network and the Molecular Complex Detection algorithm. Cox regression, meta-analysis and immunohistochemistry were applied to identify genes significantly correlated with relapse-free survival (RFS). We estimated immune infiltration using three different algorithms (TIMER 2.0, CIBERSORTx, and TIDE). Single-cell sequencing data were processed by Seurat software.

**Results:**

Poor RFS was observed in the low TME immune score groups (log-rank *P < 0.05*). EPSTI1 was demonstrated to be significantly correlated with RFS (*P < 0.05*) in stage III CC. Meta-analysis comprising 547 patients revealed that EPSTI1 was a protective factor (HR = 0.79, 95% CI, 0.65-0. 96; *P* < 0.05)). More immune infiltrates were observed in the high EPSTI1 group, especially M1 macrophage and myeloid dendritic cell infiltration (*P <* 0.05).

**Conclusion:**

The TME immune score is positively associated with better survival outcomes. EPSTI1 could serve as a novel immune prognostic biomarker for stage III CC.

## Introduction

Colorectal cancer is the third most common malignant disease and the second leading cause of cancer death in the world, with approximately 1.93 million new cases and 930,600 related deaths in 2020 (1). Tumor-node-metastasis (TNM) staging remains the key determinant of colorectal cancer prognosis and therapy. In patients with localized colon cancer (CC), the 5-year overall survival (OS) is 99% and 70% for stage I and II CC, respectively, versus only 45-65% for stage III patients (2). However, an increasing number of reports have demonstrated the wide variability of survival outcomes in stage III CC according to T and N sub-stages (3), possibly reflecting tumor heterogeneity. Prognostic assessment in stage III CC could be refined by using validated biomarkers beyond the TNM classification system.

The molecular features and prognostic value of the tumor immune microenvironment have been extensively reported in various cancer types (4, 5). The colon harbors a large number of diverse immune cells to maintain gut homeostasis. In CC, however, these cells lose their tight and well-organized modulation (6). It was found that *in-situ* immune cell infiltration in CC is associated with favorable survival (7, 8) and that reduced immune cytotoxicity and lack of T-cell infiltration in CC predict adverse outcomes (9, 10), suggesting that the tumor microenvironment (TME) might be a promising source of novel diagnostic and prognostic biomarkers.

Immunohistochemistry (IHC) and fluorescence-activated cell sorting (FACS) have long been the primary methods for assessing tumor-infiltrating immune populations. Due to the limited number of immune markers that can be measured simultaneously, these two conventional methods are incapable of demonstrating a comprehensive landscape of diverse immune cell infiltrates and do not provide sufficient resolution to discriminate closely related immune cell clusters. Recent studies have revealed that the number of various infiltrating immune cell types in a specimen can be inferred from gene expression patterns specific or abundant to a particular cell type (11, 12). Remarkably, based on accumulating transcriptomic data, several computational algorithms have been established to evaluate large-scale immune landscapes (13, 14, 15).

Based on the transcriptome data, we categorized stage III CC patients from two independent cohorts into high and low TME immune score groups. Consistent with previous studies, better survival was observed in patients with higher TME immune scores. Additional results from various public datasets confirmed that EPSTI1 was differentially expressed between the high and low TME immune score groups and that its expression was significantly associated with relapse-free survival (RFS) in patients with stage III CC. To our knowledge, few investigations have explored EPSTI1’s role in the tumor immunity of stage III CC. In this study, we revealed that more immune infiltrates, especially M1 macrophages and myeloid dendritic cells (mDCs), were found in tumors with higher EPSTI1 expression. The association between EPSTI1 and the above two immune cell types was further validated by single-cell RNA sequencing analysis, suggesting that EPSTI, as an immune biomarker, could predict the RFS in stage III CC.

## Materials and methods

### Data source

We systematically searched publicly available colon cancer datasets. Studies with no survival or TNM staging information were removed from further assessment. Five cohorts with bulk sequencing (TCGA-COAD, GSE39582, GSE37892, GSE17538, GSE14333) and one single-cell sequencing dataset (GSE178341) were enrolled. The RNA sequencing and clinical data of GSE39582, GSE37892, GSE17538, GSE14333 and GSE178341 were downloaded from the medics Gene Expression Omnibus (GEO, https://www.ncbi.nlm.nih.gov/geo/). Gene expression data and corresponding clinical information from The Cancer Genome Atlas (TCGA) colon cancer project were downloaded from the UCSC Xena browser (https://xenabrowser.net/datapages/).

Mutation data of the TCGA-COAD cohort were downloaded from the National Cancer Institute Genomic Data Commons (https://gdc.cancer.gov/about-data/publications/mc3-2017). Only TNM stage III CC patients in each dataset were included in this study. The above datasets were utilized in compliance with the ethical requirements of the GEO and TCGA projects. The study was conducted in accordance with the Declaration of Helsinki.

### Identification and verification of genes related to the TME immune score and RFS

We performed TME immune scoing for stage III CC patients in the GSE39582 and TCGA-COAD cohorts using R package “TMEscore”. The cut-off value of the TME immune score was selected by X-tile software (version 3.6.1, https://medicine.yale.edu/lab/rimm/research/software/). Based on the cut-off values, patients in both cohorts were divided into high and low TME immune score groups. We compared the difference in relapse-free survival (RFS) between the two groups using the Kaplan-Meier method. The receiver operating characteristic (ROC) curves were then plotted to assess the predictive power of the TME immune score for RFS. Genes with |log2 Fold Change| > 1 and adjusted P value < 0.05 were defined as differentially expressed genes (DEGs). The intersection of the DEGs from the GSE39582 and TCGA-COAD datasets was entered into the STRING database (https://cn.string-db.org/) to construct the protein-protein interaction (PPI) network. The network was then imported into Cytoscape software (version 3.9.0, https://cytoscape.org/), and key gene modules were identified using the Molecular Complex Detection (MCODE) algorithm. Functional and pathway enrichment analysis of key gene modules were performed in the Metascape database (https://metascape.org/gp/index.html#/main/step1).

We then used univariate Cox regression analysis to identify genes significantly associated with RFS in the above key gene modules. The association between each gene and RFS was further assessed by meta-analysis combining the datasets GSE39582 (n = 206), TCGA-COAD (n = 128), GSE14333 (n = 81), GSE17538 (n = 75) and GSE37892 (n = 57). If robust heterogeneity was not observed (I^2^ < 40%, *P* > 0.05), the fixed-effects model was chosen to pool HRs from different cohorts. Otherwise, the random-effects model was selected.

### Gene mutation analysis

In the TCGA-COAD dataset, 113 patients with stage III CC had complete somatic mutation data. In contrast, only TP53, KRAS, BRAF and mismatch repair mutations were available in the GSE39582 dataset. We compared the mutations and the tumor mutation burden (TMB) between the high and low EPSTI1 groups in the TCGA-COAD cohort. We also analyzed the mutation status of TP53, KRAS, and BRAF and microsatellite stability in both groups.

### Inference of TME immune cell infiltration

To quantify the degree of immune cell infiltration in each stage III CC sample, we applied three widely accepted algorithms for evaluation: TIMER 2.0 (http://timer.cistrome.org/), TIDE (http://tide.dfci.harvard.edu/) and CIBERSORTx (https://cibersortx.stanford.edu). According to the algorithm instructions, we uploaded the prepared gene expression matrix into the web tool to obtain infiltration scores. Only the immune cell types detected in more than 50% of the samples were included in further analysis. In the CIBERSORTx estimation procedure, we ran the web tool with LM22 gene signatures and 1000 permutations.

### Single-cell RNA sequencing data analysis

We performed scRNA-seq analysis using the R package “Seurat” (version 4.1.0). Cells with less than 200 genes and more than 50% mitochondrial counts were excluded from the analysis. The expression matrix was then normalized using the “SCTransform” function, and the top 3000 highly variable genes were subjected to principal component analysis (PCA). We constructed the shared nearest neighbor (SNN) graph and the uniform manifold approximation and projection (UMAP) embedding with the top 20 principal components. The identification of main cell types was consistent with the original literature. Based on the M1/M2 macrophage and pDC/mDC gene signatures summarized in the literature (see **Table S3**), we further subclustered the macrophages and DCs using the R package CelliD (16). The proportion of cells in each subpopulation was then calculated. The expression of M1 versus M2 up- and down-regulated genes (17), and plasmacytoid cell type DC (pDC) versus mDC up- and down-regulated genes (18) were scored using the “AddModuleScore” function. The same function was also used to compute the activity scores of immune-related signaling pathways from the Broad Institute’s Hallmark collection.

### Statistical analysis

Patient groups or cell groups were compared using Welch’s t-test if the continuous variables were normally distributed; otherwise, the Mann-Whitney U test was applied. Categorical variables were compared using the chi-square test. We plotted survival curves using the Kaplan-Meier method and used the log-rank test to compare survival differences. The predictive validity of the model was quantified by the area under the ROC curve (AUC). For correlation analysis, we calculated Pearson or Spearman correlation coefficients as indicated. A *P* value < 0.05 was considered significant. All statistical analyses were performed using R software (version 4.1.0).

## Results

### TME immune score predicts the RFS of stage III CC patients

**Figure 1:**
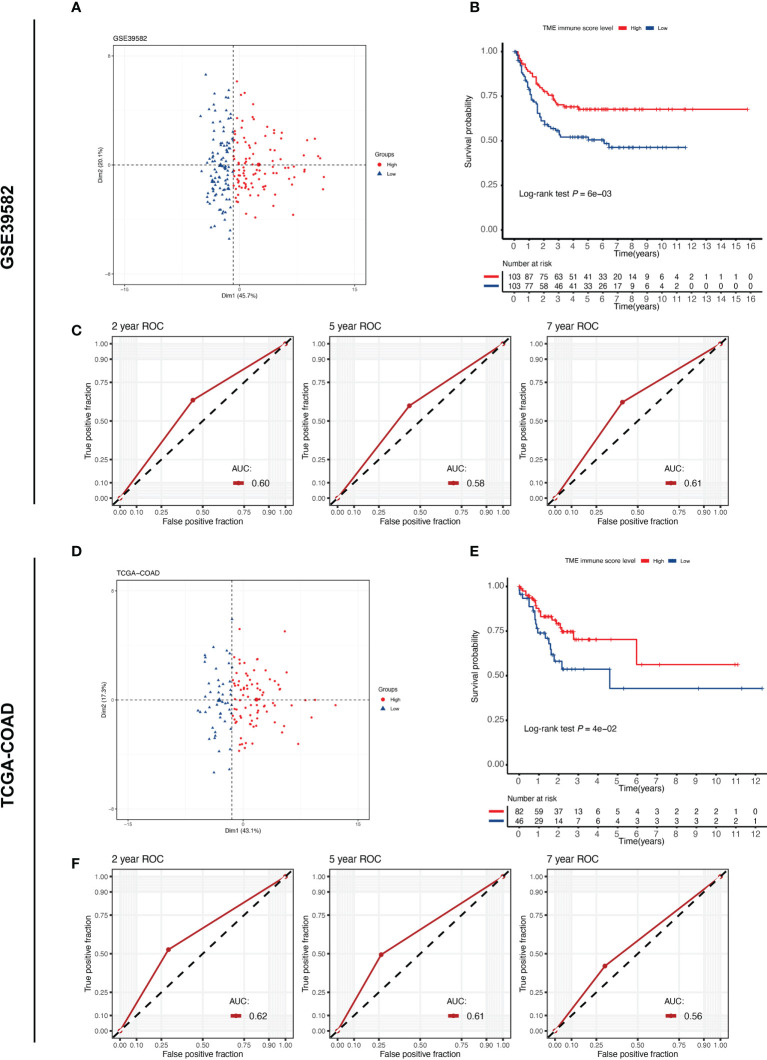
TME immune score correlates with the prognosis of patients with stage III CC. TME, tumor microenvironment. **(A, D)** PCA plot reveals different expression patterns of TME immune score-related genes in different groups of stage III CC patients from the GSE39582 and TCGA-COAD cohorts. **(B, E)** Kaplan–Meier curves of relapse-free survival according to TME immune score levels in the GSE39582 and TCGA-COAD cohorts. Stage III CC patients with high TME immune scores have a better prognosis (log-rank test *P* < 0.05) in both datasets. **(C, F)** The time-dependent ROC curves measuring the predictive power of the TME immune score on 2-, 5-, and 7-year RFS in the GSE39582 and TCGA-COAD datasets. RFS, relapse-free survival.

To investigate the relationship between the tumor immune microenvironment and RFS in stage III CC patients, we applied the TMEscore model to perform immune scoring in the GSE39582 (n = 206) and TCGA-COAD (n = 128) cohorts. The cut-off value of the TME immune score in each cohort was determined by X-tile software, and the patients were then divided into high- and low-immune score groups (**Table S1**). According to the cut-off value, 103 patients in the GSE38582 cohort were assigned to the high immune score group, and the remaining 103 patients were assigned to the low immune score group. In the TCGA-COAD cohort, the numbers were 82 and 46, respectively. The high and low TME score groups had different distribution features on the PCA dimensionality reduction map, reflecting the difference in the expression of immune-related genes between the two groups (**Figures 1 A, D**). In both cohorts, the Kaplan-Meier survival curves showed that RFS was significantly worse in the low-immune score group (**Figures 1 B, E**; log-rank *P* = 0.006 in GSE39582, log-rank *P* = 0.04 in TCGA-COAD). To measure the predictive performance of the TMEscore model, we calculated the time-dependent AUC values for both cohorts at 2, 5 and 7 years. The AUCs at these time points were 0.60, 0.58 and 0.61 in the GSE39582 cohort, while they were 0.62, 0.61 and 0.56 in the TCGA-COAD cohort (**Figures 1C, F**). These results suggest that the TME immune score can predict RFS in patients with stage III CC.

### Identification of key gene modules associated with TME immune score

**Figure 2:**
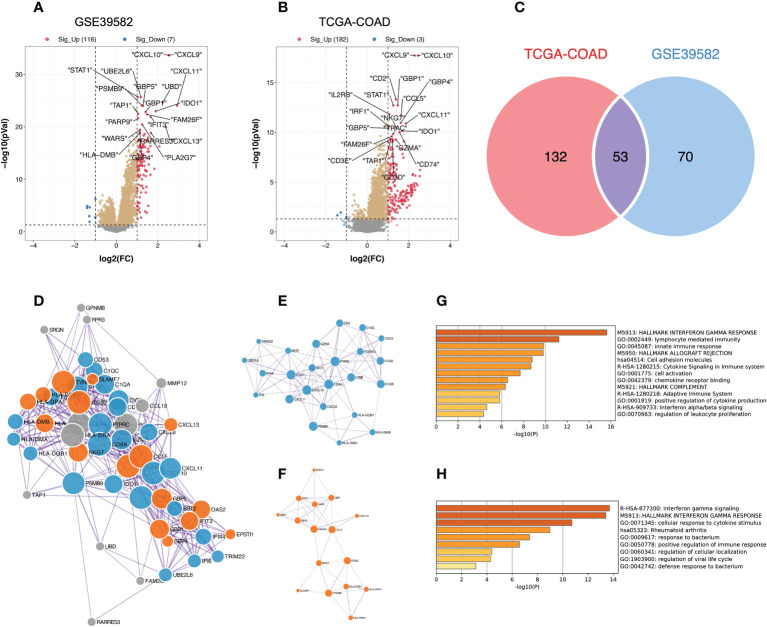
Identification of key gene modules from DEGs between the high and low TME immune score groups; DEGs, differentially expressed genes. **(A, B)** DEGs between the high and low TME immune score groups in the GSE39582 and TCGA-COAD datasets. The red dots represent significantly upregulated genes, and the blue dots represent significantly downregulated genes (adjusted *P* value < 0.05 and |log2FC| > 1); FC, fold change. **(C)** The Venn diagram reveals the intersection of DEGs in the GSE39582 and TCGA-COAD cohorts. **(D)** Protein-protein interaction network generated from the STRING database. The size of the nodes indicates the degree, which denotes the number of how many interactions (at the score threshold) that a protein has on the average in the network. The thickness of the edge indicates the combined score, which represents the confidence of the link between two proteins. **(E, F)** Critical sub-network components in the PPI network identified by the MCODE algorithm. MCODE, Molecular Complex Detection; PPI, protein-protein interaction. **(E)** MCODE cluster 1; **(F)** MCODE cluster 2. **(G, H)** Bar graph of enriched pathways across gene lists in MCODE cluster 1 and 2, colored by *P* values.

We then analyzed the DEGs between the different TME immune scoring groups in the GSE39582 and TCGA-COAD datasets. Compared to the low TME immune score group, patients in the high TME immune score group had 116 genes that were significantly upregulated and 7 genes that were significantly downregulated in GSE39582 (**Figures 2A**, **Table S2**), while 182 genes were upregulated and 3 genes were downregulated in the high immune score group in TCGA-COAD (**Figure 2B**). We then intersected the DEGs from the two cohorts to obtain 53 common genes (**Figure 2C**). By entering these genes into the STRING database, we constructed a protein-protein interaction network (**Figure 2D**). The minimum required interaction score was set as medium confidence (0.400). The PPI network was then imported into Cytoscape software, and two key gene modules were identified by the MCODE algorithm: MCODE cluster 1 contained 24 genes (**Figure 2E**), while MCODE cluster 2 contained 16 genes (**Figure 2F**). The enrichment analysis suggests that these genes are mainly related to human immune function, particularly the interferon gamma signaling pathway (**Figure 2G, H**).

### Genes associated with TME immune scores and RFS

**Figure 3:**
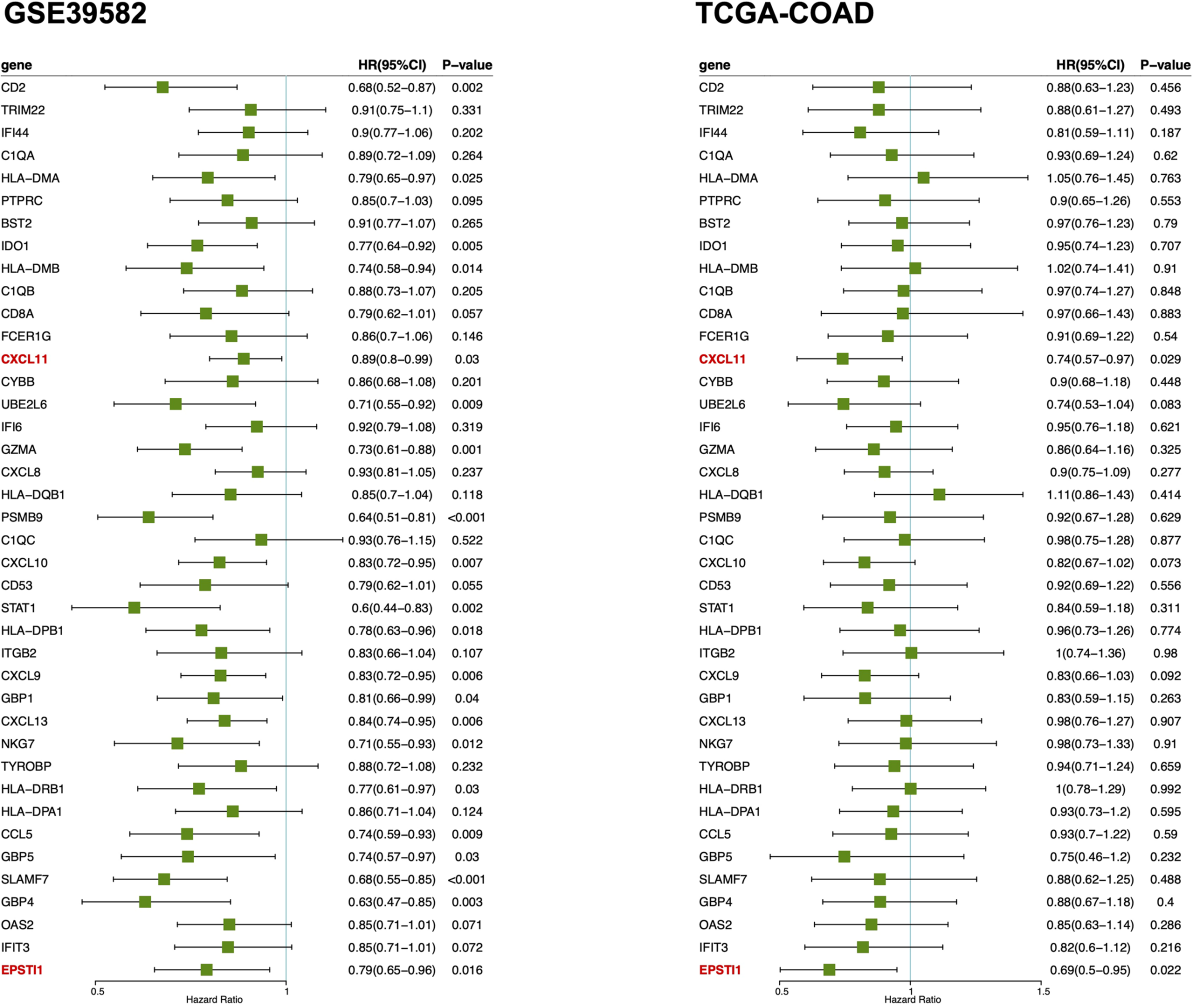
Forest plot of univariate Cox regression analysis for MCODE cluster 1 and 2. The results show that only the ESPTI1 and CXCL 11 are significant protective factors (HR < 1, *P* < 0.05) for RFS in both the GSE39582 and TCGA-COAD cohorts. MCODE, the Molecular Complex Detection algorithm; RFS, relapse-free survival.

**Figure 4:**
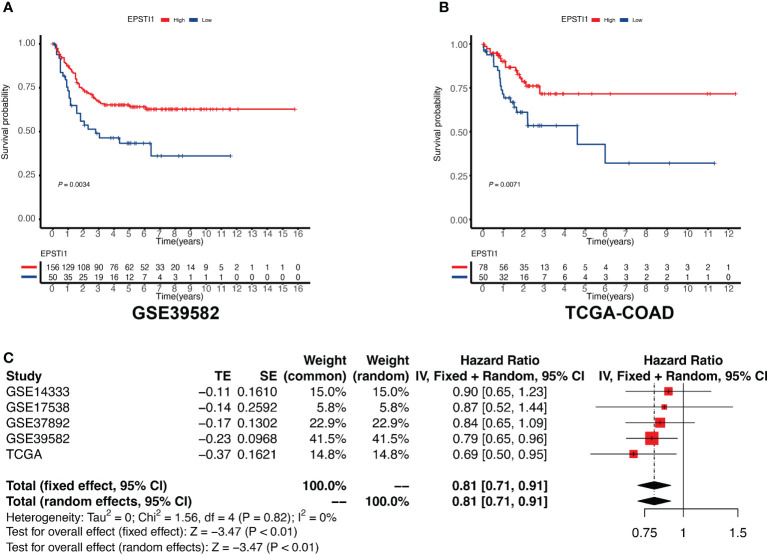
Association between EPSTI1 expression level and RFS in patients with stage III CC. CC, colon cancer; RFS, relapse-free survival. **(A, B)** Kaplan-Meier survival curves for high and low EPSTI1 groups in the GSE39582 and TCGA-COAD cohorts. Patients with high EPSTI1 levels have significantly better RFS than those with low EPSTI1 levels. **(C)** A meta-analysis of 5 independent studies shows that the expression level of EPSTI1 is a protective factor for RFS (fixed model effect, pooled HR = 0.81, 95% CI 0.71-0.91). HR, hazard ratio; CI, confidence interval.

Survival analysis was performed *via* univariate Cox regression to identify a subset of genes closely related to RFS in the MCODE clusters. The results revealed that only EPSTI1 and CXCL11 were significantly associated with RFS in both the GSE39582 and TCGA-COD cohorts (**Figure 3**). In GSE39582, the hazard ratios (HRs) for EPSTI1 and CXCL11 were 0.79 (95% CI, 0.65-0.96; *P* < 0.05) and 0.89 (95% CI, 0.80-0.99; *P* < 0.05), respectively. In TCGA-COAD, the HRs for the two genes were 0.69 (95% CI, 0.50-0.95; P < 0.05) and 0.74 (95% CI, 0.57-0.97; P < 0.05), respectively. Using X-tile software, we determined the cut-off values for EPSTI1 and CXCL11 expression levels, respectively, and divided patients in the GSE39582 and TCGA-COAD cohorts into groups with high and low expression levels of the corresponding genes. Kaplan-Meier survival curves showed that the high EPSTI1 group had prolonged RFS (**Figure 4A, B**; *P* < 0.05), and the high CXCL11 group also had a better prognosis (Supplementary **Figure 1A, B**; *P* < 0.05). To further confirm the relationship between these two genes and RFS in patients with stage III CC, 547 patients from five datasets, GSE39582 (n = 206), TCGA-COAD (n = 128), GSE14333 (n = 81), GSE17538 (n = 75) and GSE37892 (n = 57), were subjected to meta-analysis. No significant heterogeneity was observed among these datasets (*I* ^2^ < 40%, *P* > 0.05), and thus, the fixed effects model was selected for the meta-analysis. The pooled HRs of EPSTI1 and CXCL11 were 0.81 (95% CI, 0.71-0.91) and 0.92 (95% CI, 0.86-0.98), respectively (**Figure 4C**; **Supplementary Figure 1C**). These results suggest that high levels of EPSTI1 and CXCL11 expression in stage III CC are significantly associated with prolonged RFS.

To date, a wide range of studies have explored the role of CXCL11 in antitumor immunity in diverse tumors, including CC (19, 20). However, the role of EPSTI1 in antitumor immunity in CC has not yet been reported. Therefore, in the following analysis, we focused on the association of EPSTI1 with the immune microenvironment of CC and its impact on RFS.

### Mutations in the high and low EPSTI1 groups

The top 20 mutated genes in the high and low EPSTI1 groups from the TCGA-COAD are illustrated in Supplementary **Figure 2A**. Although the mean TMB was greater in the high EPSTI1 group, there was no significant difference between the two groups (**Supplementary Figure 2B**; *P* > 0.05). The mutation rate of TP53 exceeded 50% in both groups, but no significant difference was observed (**Supplementary Figures 2C, D**). The proportions of patients with BRAF mutation or microsatellite instability were also not significantly different between the two groups (**Supplementary Figures 2C, D**). In the GSE39582 cohort, the KRAS mutation rate in the high EPSTI1 group was 37%, which was significantly lower than the 61% rate in the low EPSTI group (**Supplementary Figure 2D**). Although the KRAS mutation rate in the high EPSTI1 group was still smaller than that in the low EPSTI group in the TCGA-COAD cohort (33% vs. 41%), the difference was not statistically significant (**Supplementary Figure 2C**).

### Association between EPSTI1 and immune infiltrates

**Figure 5:**
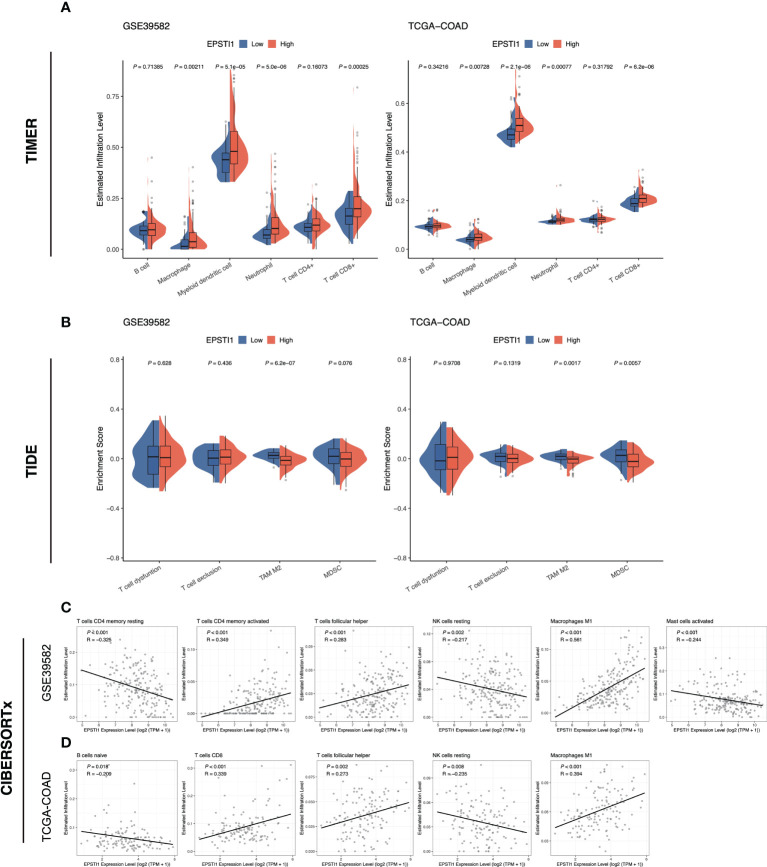
The association between EPSTI1 expression level and immune cell infiltration. **(A)** Comparison of immune cell infiltration in the high and low EPSTI1 groups estimated with the TIMER algorithm in the GSE39582 and TCGA-COAD cohorts. TIMER, Tumor Immune Estimation Resource. **(B)** Enrichment scores of immunosuppressive cell signatures estimated by the TIDE algorithm. TIDE, Tumor Immune Dysfunction and Exclusion. **(C, D)** Scatter plots of EPSTI1 expression and immune cell infiltration levels, which were estimated by the CIBERSORTx algorithm. The Pearson’s correlation coefficient (R) and corresponding P-value are shown at the left top of each plot.

In this study, we evaluated immune infiltration in tumor tissue based on bulk RNA-seq data using three different approaches, including TIMER 2.0, TIDE and CIBERSORTx. TIMER 2.0 analysis demonstrated that macrophages, myeloid dendritic cells, neutrophils and CD8 T cells were more abundantly infiltrated in the high EPSTI1 group (**Figure 5A**; *P* < 0.05). TIDE found significantly lower infiltration abundance of M2 tumor-associated macrophages (TAM) in the high EPSTI1 group in both cohorts when assessing immunosuppressive cells (**Figure 5B**; *P* < 0.05). For MDSCs, the infiltration abundance was significantly lower in the high EPSTI1 group in the TCGA-COAD cohort (**Figure 5B**; *P* < 0.05). In the GSE39582 cohort, the difference was only marginally significant (**Figure 5B**; P = 0.076), although the high EPSTI1 group still had a smaller infiltration abundance than the low EPSTI1 group. The CIBERSORTx algorithm calculates the infiltration status of 22 types of immune cells. In this study, we excluded a cell type if its infiltrative abundance was calculated to be zero in more than 50% of the samples. Pearson correlation analysis revealed that in the GSE39582 cohort, EPSTI1 expression was positively correlated with the infiltration of memory activated CD4 T cells, follicular helper T cells, and M1 macrophages (**Figure 5C**; R > 0.2, *P* < 0.05), and negatively correlated with the infiltration of resting memory CD4 T cells, resting NK cells and activated mast cells (**Figure 5C**; R < -0.2, *P* < 0.05). In the TCGA-COAD cohort, EPSTI1 expression was positively correlated with the infiltration of CD8 T cells, follicular helper T cells and M1 macrophages (**Figure 5D**; R > 0.2, *P* < 0.05), and negatively correlated with the infiltration of naive B cells and resting NK cells (**Figure 5D**; R < -0.2, *P* < 0.05). Notably, M1 macrophages displayed a strong correlation with EPSTI1 expression in both cohorts (R = 0.561 in GSE39582; R = 0.394 in TCGA-COAD).

### Expression of EPSTI1 at the single-cell level

**Figure 6:**
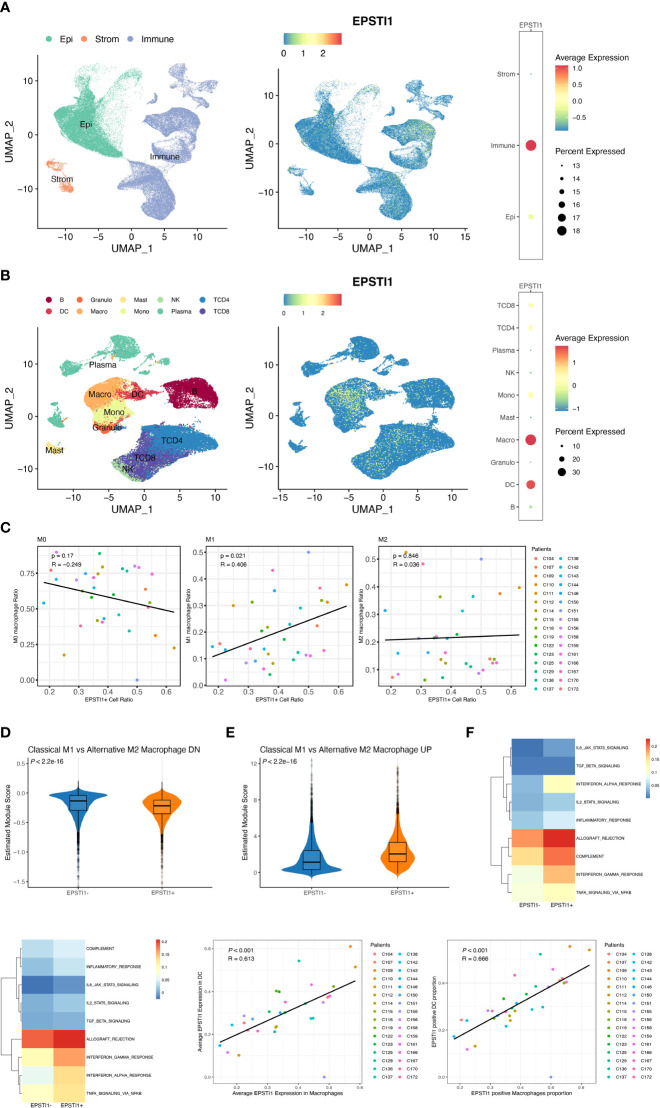
The analysis of EPSTI1 expression at the single cell level. **(A, B)** EPSTI1 expression levels in various cell types revealed by dimensional reduction plots (left and middle panels) and dot plots (right panels). The results show that EPSTI1 is mainly expressed in immune cells, especially in macrophages and dendritic cells. Epi, epithelial cells; Strom, stromal cells; B, B cells; Granulo, granulocytes; Mast, mast cells; NK, natural killer cells; TCD4, CD4 positive T cells; TCD8, CD8 positive T cells; DC, dendritic cells; Macro, macrophages; Mono, monocytes; Plasma, plasma cells. **(C)** The EPSTI1+ macrophage ratio and M1 macrophage ratio are significantly correlated (R = 0.406, *P* = 0.021) **(D, E)** The module scores of gene signatures related to M1/M2 polarization in EPSTI1+/- macrophages suggest that EPSTI1+ macrophages have more M1 features. **(F)** Mean pathway activity scores of EPSTI1+/- macrophages. The immune-related pathways appear to be more active in EPSTI1+ macrophages. **(G)** Mean pathway activity scores of EPSTI1+/- DCs. Immune-related pathways are scored higher in EPSTI1+ DCs. **(H, I)** Macrophages and DCs are significantly correlated (R > 0.60, *P* < 0.001) in terms of EPSTI1 expression levels and the proportion of EPSTI1 positive cells.

In the bulk RNA-seq datasets above, EPSTI1 was closely associated with immune infiltrates. To further investigate the relationship between EPSTI1 and tumor immune cells, we analyzed 32 stage III CC patients from the single-cell dataset GSE178341. According to the quality control criteria in the original literature, we obtained 114,928 cells from CC tissues. Cell types were identified with reference to the original paper. From the EPSTI1 dimplot and dotplot (**Figure 6A**), we found that EPSTI1 was predominantly expressed in immune cells, both in terms of EPSTI1 expression levels and the proportion of EPST1-positive cells. Further sorting of immune cells revealed that macrophages and DCs were the main cell clusters expressing EPSTI1 (**Figure 6B**), similar to the immune infiltration analysis of the bulk RNA-seq datasets above.

Several studies have indicated that M1macrophages exert tumor-preventing activities, whereas M2 macrophages are associated with immunosuppression (21, 22, 23). At the single-cell level, the ratio of EPSTI1+ macrophages was significantly correlated with that of M1 macrophages (R = 0.405, *P* = 0.022; **Figure 6C**, **Supplementary Figure 3A**), which was consistent with the CIBERSORTx analysis of bulk sequencing described previously. Moreover, EPSTI1^+^ macrophages scored significantly lower on the M2 but significantly higher on the M1 signature modules (**Figures 6D, E**; *P* < 0.05). In the scoring of cell signaling pathway activity, EPSTI1^+^ macrophages had a higher mean score for immune-related signaling pathways (**Figure 6F**), showing a different function pattern from EPSTI1^-^ macrophages.

DCs were another major cell cluster that expressed EPSTI1 in our study. By origin, DCs can be classified into mDCs, which are derived from common myeloid progenitors (CMPs) that also produce macrophages, and pDCs, which are derived from common lymphocyte progenitors (CLPs) that also produce B cells, T cells and NK cells. Although the proportion of EPSTI1+ DCs was positively correlated with that of mDCs without statistical significance (R = 0.302, P = 0.093; **Supplementary Figure 3B, C**), EPSTI1^+^ DCs had significantly lower pDC but higher mDC feature scores (**Supplementary Figures 3D, E**; *P* < 0.05). Similar to EPSTI1^+^ macrophages, the mean scores of immune-related signaling pathways were higher in EPSTI1+ DCs (**Figure 6G**). The association between DCs and macrophages in EPSTI1 expression was also inspected by Pearson correlation analysis. In the 32 patients with stage III CC in GSE178341, macrophages and DCs were significantly correlated (R > 0.6, *P* < 0.05), both in terms of the average level of EPSTI1 expression and the proportion of EPSTI1^+^ cells (**Figures 6H, I**).

## Discussion

The immunocyte infiltration has received extensive attention for its important role in both tumor prognosis and therapy (24, 25). In our study, patients with a high TME immune score, which reflects immunocyte infiltration, experienced better RFS. The result was consistent with previous reports using Immunoscore calculated by assessing CD3 and CD8 immunohistochemical staining both in the tumor center and invasive margin (26). To explore more potential molecules associated with both immunocyte infiltration and prognosis, we compared the DEGs between the high and low TME immune score groups and screened out EPSTI1 and CXCL11. However, CXCL11 has been included in the TME immune score algorithm and widely investigated in various cancers, including colon cancer (20, 27). EPSTI1, initially identified as an induced gene in a three-dimensional tumor environment assay (28), was reported to promote epithelial-mesenchymal transition (EMT) and tumor metastasis in breast cancer (29, 30, 31). Its significance in colon cancer, especially its participation in the immune response, has not been well explored.

To further confirm the prognostic significance of EPSTI1 in stage III CC, we explored the relationship between RFS and EPSTI1 expression in a meta-analysis including more than 500 patients in 5 datasets at the mRNA level. The results indicated that high expression of EPSTI1 was significantly associated with better RFS. Analysis of the single-cell dataset further showed that the average expression of EPSTI1 was highest in immune cells.

The types and functions of infiltrative immune cells are various and complex. Analysis results from both bulk tissue and single-cell RNA sequencing revealed that the expression of EPSTI1 was significantly high in macrophages and DCs, especially classically activated M1 macrophages, playing roles in antitumor immunity (32, 33, 34). In our analysis of pathway activity assessment for macrophages and DCs, EPSTI1 was found to be associated with several immune-related pathways, such as interferon-γ (IFNγ) response, interleukin-6 (IL6)-Jak-Stat3 signaling and tumor necrosis factor-α (TNF-α) signaling activated by nuclear factor κB (NFκB). M1 phenotypes of macrophages are usually polarized *via* IFNγ, and subsequently release numerous cytokines (such as TNF-α and IL-6) and reactive oxygen/nitrogen species to realize the tumoricidal activity (35). An NFκB-dependent and IFNγ-regulated gene network in mDCs promotes antigen presentation from dying tumor cells and the subsequent recruitment and activation of cytotoxic T cells (36). In Kim YH et al.’s research, EPSTI1 was found to be highly expressed in macrophages exposed to IFNγ and lipopolysaccharide (LPS) and to modulate M1 polarization *via* the Stat1 and p65 pathways (37). Therefore, we speculate that EPSTI1 in the stage III CC exerts antitumor immunity and inhibits tumor progression by promoting macrophage and mDC infiltration, accelerating the M1 polarization of macrophages, enhancing the antigen presentation ability of M1 macrophages and DCs and reinforcing subsequent tumor killing.

Apart from chemotherapy, radiotherapy and targeted therapy, immunotherapy has been emerging as another pillar for tumor treatment (38, 39). Although immune checkpoint blockers (ICBs), which primarily target cytotoxic T lymphocytes, are widely used in current clinical practice (40, 41), TAMs and DCs have also been favored and explored in recent decades. A variety of therapeutic strategies targeting TAMs and DCs are being tested in basic researches and clinical trials (42, 43, 44), for example, inhibiting mononuclear macrophage recruitment, TAM depletion and inhibition of activation, reprogramming TAMs and DC-based cancer vaccines. Correspondingly, a variety of relevant molecules are being targeted, for instance, blockade of CD47 to enhance the phagocytotic abilities of antigen-presenting cells, inhibition of phosphoinositide 3-kinase γ (PI3Kγ) to interrupt M2 polarization, and Toll-like receptor (TLR) agonists to induce M1 polarization. Considering the potential significance in macrophages and DCs, EPSTI1 deserves more in-depth research and might be another target for cancer immunotherapy.

In conclusion, the TME immune score is positively associated with better survival outcomes. EPSTI1 could serve as a novel immune prognostic biomarker for stage III CC.

## Data availability statement

The original contributions presented in the study are included in the article/**Supplementary Material**. Further inquiries can be directed to the corresponding authors.

## Ethics statement

The studies involving human participants were reviewed and approved by The Institutional Review Board of Xiangya Hospital. The patients/participants provided their written informed consent to participate in this study.

## Author contributions

Conception and design: XW, ZZ, QP. Data acquisition: XW, QP. Data analysis/interpretation: XW, WC, XZ, XD, QP. Writing of original draft: XW, WC, ZZ, QP. All authors contributed to the article and approved the submitted version.

## Funding

This work was supported by China Postdoctoral Science Foundation: 2020M670103ZX; and the Construction of Innovative Ability of National Clinical Research Center for Geriatric Disorders (no. 2019SK2335).

## Conflict of interest

The authors declare that the research was conducted in the absence of any commercial or financial relationships that could be construed as a potential conflict of interest.

## Publisher’s note

All claims expressed in this article are solely those of the authors and do not necessarily represent those of their affiliated organizations, or those of the publisher, the editors and the reviewers. Any product that may be evaluated in this article, or claim that may be made by its manufacturer, is not guaranteed or endorsed by the publisher.

## Supplementary material

Supplementary Figure 1 Identification of RFS-related genes in MCODE cluster 1 and 2. **(A)** Forest plot of univariate Cox regression analysis for MCODE cluster 1 and 2. The results show that only the ESPTI1 and CXCL 11 are protective factors (HR < 1, P < 0.05) for RFS in both the GSE39582 and TCGA-COAD cohorts. MCODE, the Molecular Complex Detection algorithm; RFS, relapse-free survival. **(B, C)** Kaplan-Meier survival curves for high and low CXCL11 groups in the GSE39582 and TCGA-COAD cohorts. Patients with high CXCL11 levels have significantly better RFS than those with low CXCL11 levels. **(D)** A meta-analysis of 5 independent studies shows that the expression level of CXCL11 is a protective factor for RFS (fixed model effect, pooled HR = 0.92, 95% CI 0.86-0.98). HR, hazard ratio; CI, confidence interval.

Supplementary Figure 2 Analyses of somatic mutation profiles in stage III CC patients. **(A)** Oncoplot of detailed mutation information of top 20 genes in low and high EPSTI1 groups of the TCGA-COAD cohort. Genes are ordered by their mutation frequency. **(B)** Comparison of tumor mutation burden between low and high EPSTI1 groups in the TCGA-COAD cohort. **(C, D)** Stacked bar plot shows the distribution of mutation spectra for low and high EPSTI1 groups in the TCGA-COAD and GSE39582 cohorts.

Supplementary Figure 3 Supplementary analysis of EPSTI1 expression at the single-cell level. **(A, B)** Subclusters of macrophages and DCs identified by the R package CelliD. **(C)** The proportion of EPSTI1+ cells is positively correlated with that of mDCs without statistical significance (R = 0.302, *P* = 0.093). **(D, E)**The module scores of gene signatures related to plasmacytoid/myeloid DC. The results show that EPSTI1+ DCs have more myeloid features.
