# Supplementary figures and images for: EPSTI1 as an immune biomarker predicts the prognosis of patients with stage III colon cancer
Source: Front Immunol. 2022 Oct 18;13:987394. doi: 10.3389/fimmu.2022.987394 (PMC9623419; doi:10.3389/fimmu.2022.987394)

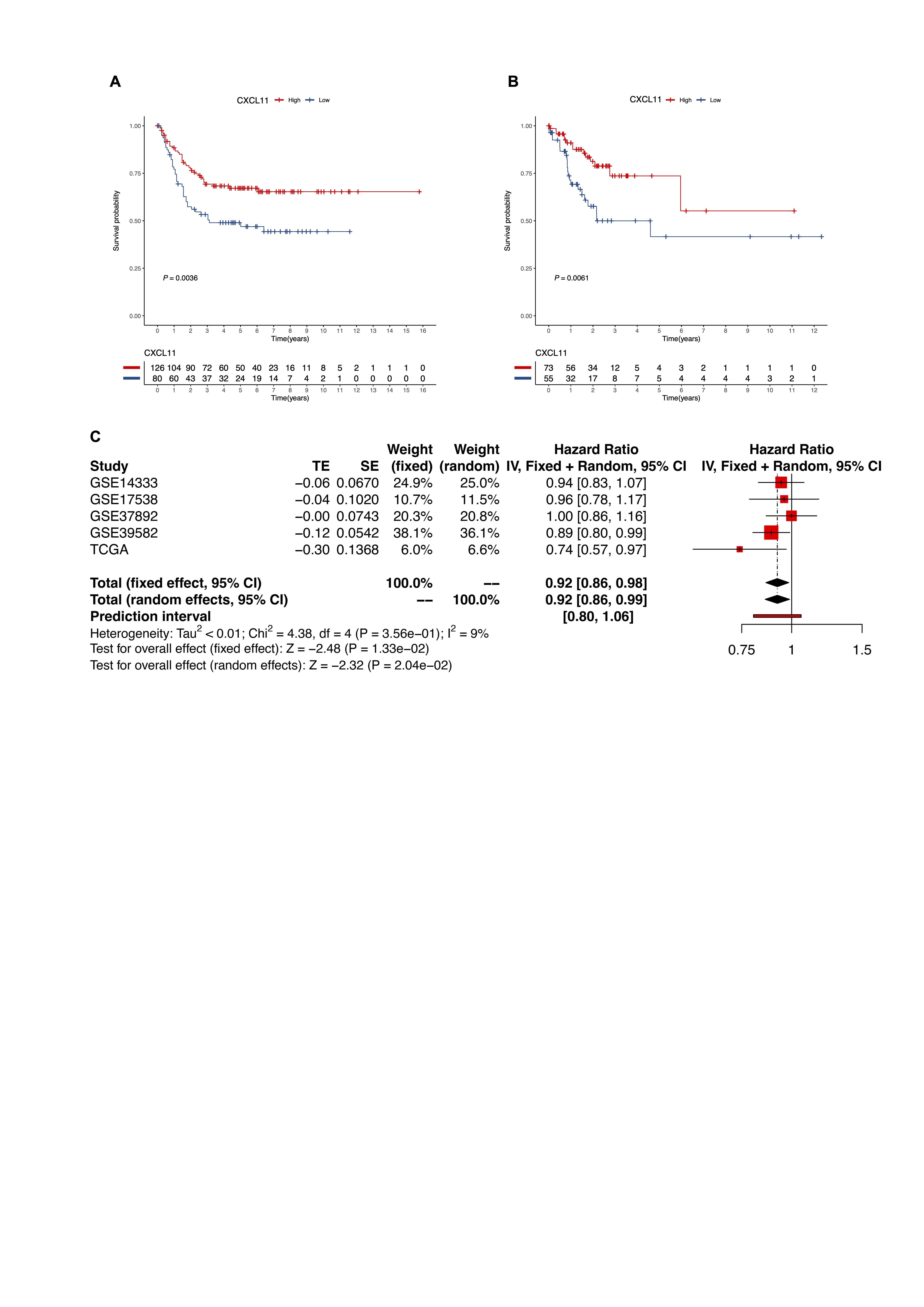

Supplement: Supplementary Figure 1 — Identification of RFS-related genes in MCODE cluster 1 and 2. (A) Forest plot of univariate Cox regression analysis for MCODE cluster 1 and 2. The results show that only the ESPTI1 and CXCL 11 are protective factors (HR < 1, P < 0.05) for RFS in both the GSE39582 and TCGA-COAD cohorts. MCODE, the Molecular Complex Detection algorithm; RFS, relapse-free survival. (B, C) Kaplan-Meier survival curves for high and low CXCL11 groups in the GSE39582 and TCGA-COAD cohorts. Patients with high CXCL11 levels have significantly better RFS than those with low CXCL11 levels. (D) A meta-analysis of 5 independent studies shows that the expression level of CXCL11 is a protective factor for RFS (fixed model effect, pooled HR = 0.92, 95% CI 0.86-0.98). HR, hazard ratio; CI, confidence interval. [file Image_1.jpeg]

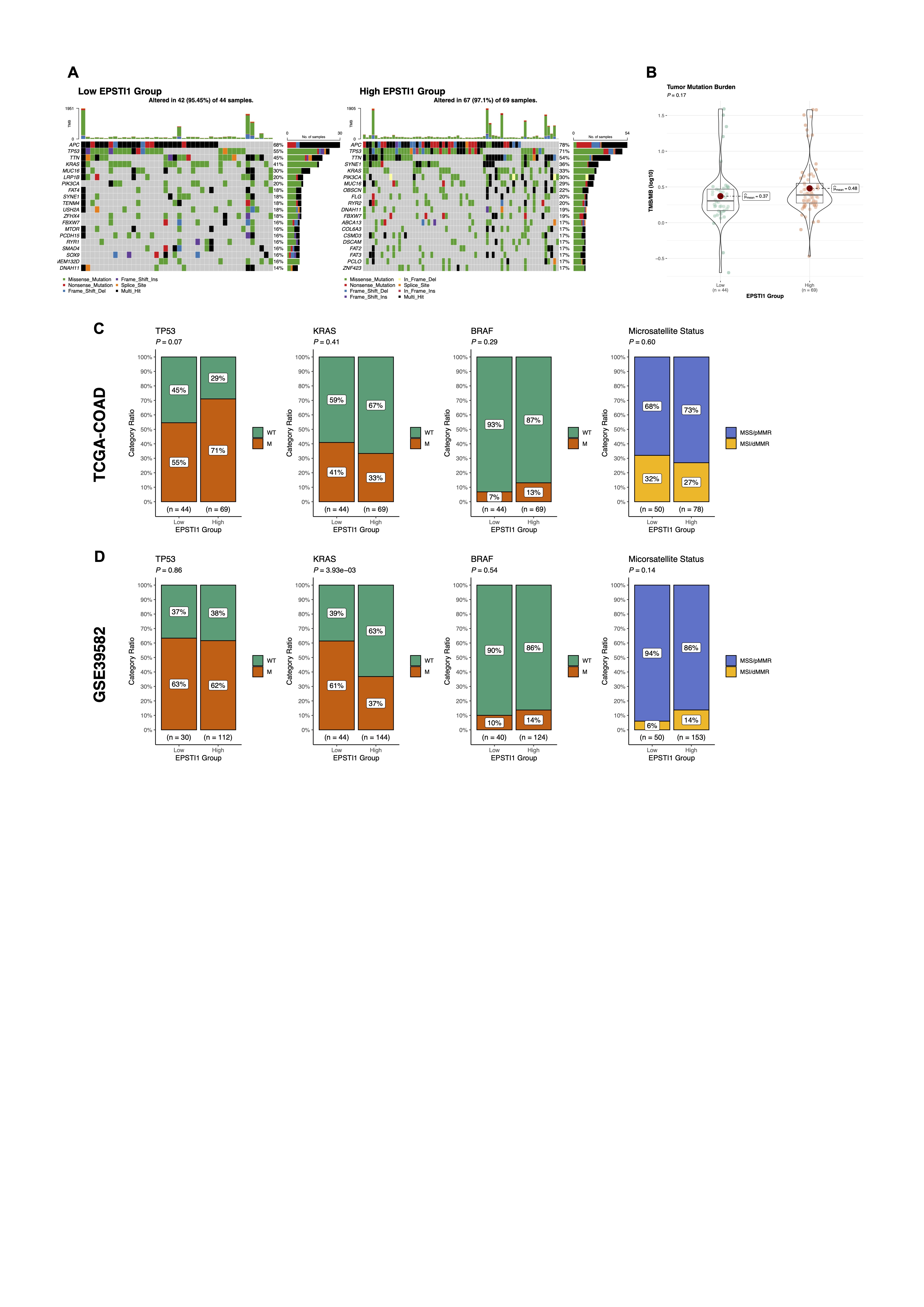

Supplement: Supplementary Figure 2 — Analyses of somatic mutation profiles in stage III CC patients. (A) Oncoplot of detailed mutation information of top 20 genes in low and high EPSTI1 groups of the TCGA-COAD cohort. Genes are ordered by their mutation frequency. (B) Comparison of tumor mutation burden between low and high EPSTI1 groups in the TCGA-COAD cohort. (C, D) Stacked bar plot shows the distribution of mutation spectra for low and high EPSTI1 groups in the TCGA-COAD and GSE39582 cohorts. [file Image_2.jpeg]

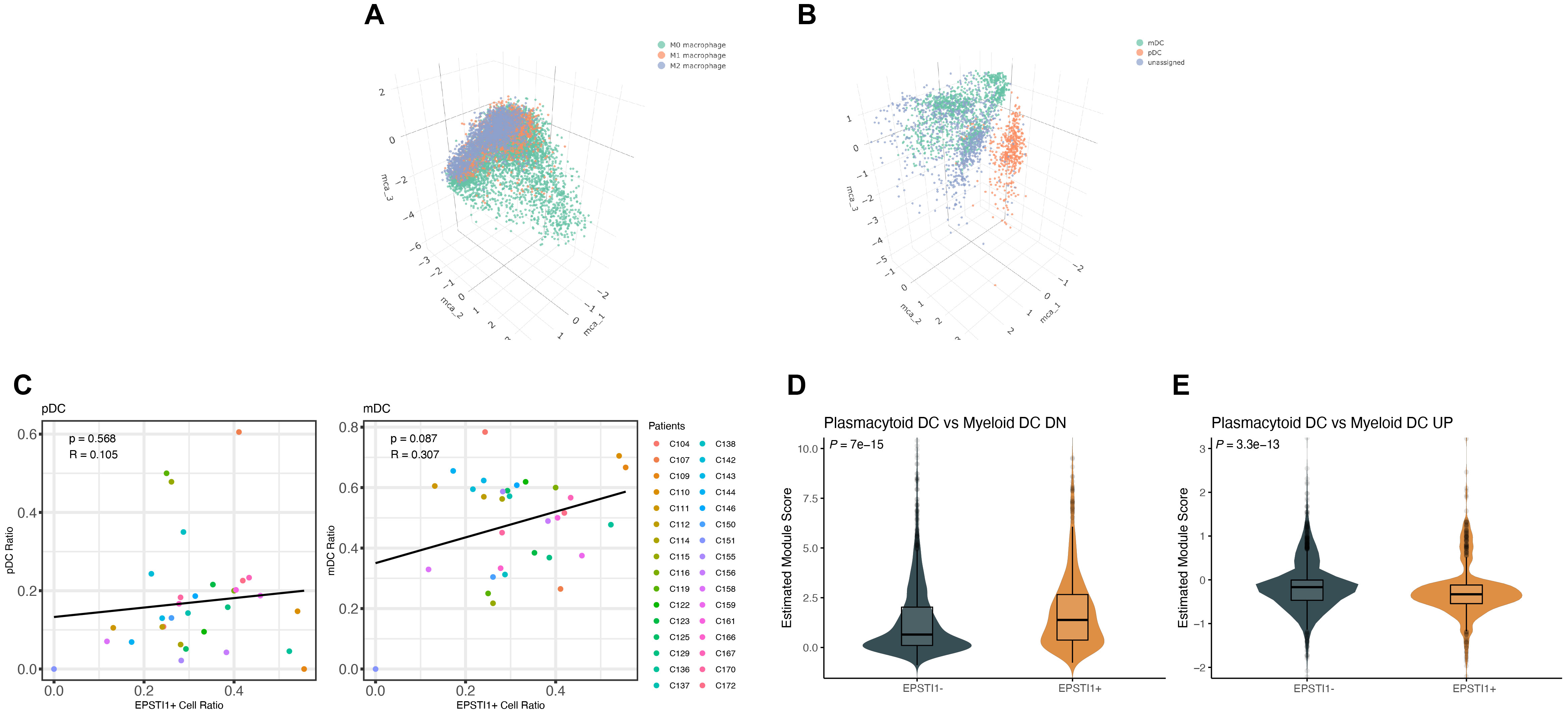

Supplement: Supplementary Figure 3 — Supplementary analysis of EPSTI1 expression at the single-cell level. (A, B) Subclusters of macrophages and DCs identified by the R package CelliD. (C) The proportion of EPSTI1+ cells is positively correlated with that of mDCs without statistical significance (R = 0.302, P = 0.093). (D, E)The module scores of gene signatures related to plasmacytoid/myeloid DC. The results show that EPSTI1+ DCs have more myeloid features. [file Image_3.jpeg]
